# Effect of Selenium on Thyroid Disorders: Scientometric Analysis

**Published:** 2019-03

**Authors:** Farzad PAKDEL, Roghayeh GHAZAVI, Roghayeh HEIDARY, Athena NEZAMABADI, Maryam PARVIZI, Mahsa HAJI SAFAR ALI MEMAR, Reza GHAREBAGHI, Fatemeh HEIDARY

**Affiliations:** 1. Department of Oculofacial, Eye Research Center, Farabi Hospital, Tehran University of Medical Sciences, Tehran, Iran; 2. Vice Chancellery of Research and Technology, Isfahan University of Medical Sciences, Isfahan, Iran; 3. International Virtual Ophthalmic Research Center, Tehran, Iran; 4. Department of Pathology, Mofid Children’s Hospital, Shahid Beheshti University of Medical Sciences, Tehran, Iran; 5. Immunoregulation Research Center, Shahed University, Tehran, Iran; 6. Department of Ophthalmology, Poostchi Ophthalmology Research Center, Shiraz University of Medical Sciences, Shiraz, Iran

**Keywords:** Selenium, Thyroid disorders, Scientometric analysis, Field maps, International collaborations

## Abstract

**Background:**

Association of Selenium (Se) deficiency, an essential trace element, has been found with human diseases. Identifying literature trends on the effects of Se on the thyroid may guide in planning future studies.

**Methods:**

A literature search was conducted using the Web of Science database to identify studies on Se and the thyroid published over the 20 years duration (1995–Dec 2014). Scientometric indices were used to draw field maps. The scientific processes, structure, evidence history, and international collaborations were included in the map. The most influential authors, journals, institutions, and countries were also examined.

**Results:**

Our search identified 184 research and review papers. The number of scientific studies on Se and the thyroid has been irregular, but interest in this topic has increased in recent years. The highest number of studies was published in 2014 (16 papers) and overall growth factor of publication was 3.78. Overall, 744 authors from 282 institutions in 43 countries published in this field. The author J. Kohrle (828 citations, 14 publications), and German scientists (1272 citations, 30 publications) were most influential.

**Conclusion:**

This study reveals the interrelationships between different publications on the effects of Se on the thyroid. Leading scientific issues and their extent of impact were successfully determined by examining citations. The results of systematic citations and mapping fields can be used to assist in policy and management contexts.

## Introduction

Selenium (Se) is a rare mineral that is essential to the human body. Selenium deficiency has been found to predispose for and lead to numerous diseases, such as cancer, impaired immune function, neurodegenerative age-related disorders, and thyroid hormone axis disturbances. The human thyroid gland has a very high Se content per mass unit, like other endocrine organs and the brain (1–4). Selenium affects both benign and malignant thyroid diseases. In Hashimoto’s thyroiditis, T-cell associated thyroid destruction (TH1-induced) results in hypothyroidism. In Graves’ disease, hyperstimulation of the thyroid by autoantibodies (TH2-induced) results in hyperthyroidism.

An adequate nutritional supply of Se, iodine, and iron are needed to maintain a healthy thyroid during childhood, adolescence, adulthood, and old age (5). Humans obtain Se from their diet as selenomethionine (SeMet), selenocysteine, and sulfur amino acid analogs, found in vegetables and animal products. Animal meats and seafood are particularly rich in Se. Populations in numerous areas of the world are susceptible to Se and iron insufficiencies because of low soil content of these minerals, resulted from erosion and glacial washout. An inadequate intake of dietary Se, defined as 3–22 microgram per day (μg/day), is associated with serious endemic diseases, including myxedematous cretinism, osteoarthropathy, and cardiomyopathy. However, the onset of these diseases requires the presence of additional factors, other than Se deficiency, including a diet rich in goitrogenic foods, bacterial or viral infection, and fungal pollutants found in wheat (2).

Excess reactive oxygen species and hydrogen peroxide are produced by thyroid follicles during biosynthesis of thyroid hormones. Selenoproteins, including glutathione peroxidase (GPx) and thioredoxin reductase, play a role in protecting the thyroid gland with cellular antioxidative defense systems and redox control. In cases of severe Se deficiency, the lack of GPx activity may contribute to oxidative damage of thyroid cells and result in thyroid damage and fibrosis. Myxedematous cretinism was associated with serious and constant selenium deficiency which leads to impaired thyroid hormone biosynthesis and intensifies follicle destruction and replacement with fibrotic tissue (1).

The identification of selenocysteine-containing proteins has discovered the relationships between Se and iodine and the hormone network. Several selenoproteins help protect thyrocytes from hydrogen peroxide produced during thyroid hormone biosynthesis (3). Selenium is important for maintaining thyroid homeostasis and may impact on the natural history of thyroid diseases, including autoimmune thyroiditis (AIT) (2). In fact, current clinical trials demonstrated that selenium treatment was effective against serum concentrations of antithyroid peroxidase in patients with Hashimoto’s thyroiditis (6). A number of other trials with dietary supplements included subjects with a variety of pathologies have been performed. These demonstrated the positive effects of increasing Se dietary intake. In addition, a current prospective, controlled, blinded study showed promising results of Se-dependent treatments on postpartum thyroiditis and hypothyroidism, a common and serious complication in pregnancy associated with autoimmune thyroid disease (AITD). Epidemiological analyses have shown that a large fraction of goiters are preventable by improving Se intake in endemic areas, provided that an adequate iodine supply is established before Se intervention. Furthermore, Se supplementation was recently shown to be effective in treating two forms of AITD (7).

Selenium supplementation seems promising for improving GPx and other selenoprotein activity in many thyroid disorders. The usefulness of Se therapies largely requires the bioavailability of compounds used. Therefore, SeMet may be useful for more long-term Se therapies because of its excellent bioavailability and low toxicity (8).

Several different thyroid disorders related to Se deficiency have been evaluated and effects of dietary supplements of Se have been examined (9–12), but a comprehensive study on scientometric analysis in this field is needed to identify factors that affect outcomes in this field of research, Se treatment advances, and emerging trends. Scientometric analyses can identify gaps in all research fields, allowing us to better understand what types of studies are needed. Careful science policy planning can help eliminate these gaps by focusing studies on specific questions. Furthermore, knowing the trends of scientific study in a specific area and determining the status of current research allows future research studies to be better planned. To the best of our knowledge, few scientometric studies on Se and the thyroid have been reported.

Here, we performed such an analysis to better understand what studies on Se and thyroid disorders are needed.

## Methods

Scientific publications on Se and the thyroid were searched in the ISI-Web of Science Core Collection. The literature search was restricted to two major indices, the Social Science Citation Index and the Science Citation Index (SCI) Expanded. The search was performed using free keyword searches in the title to retrieve the entire body of literature on thyroid disorders (e.g., thyroiditis, hyperthyroidism, hypothyroidism), and all forms of the Se element (e.g., selenoprotein, selenium, and Se). Keywords were searched only on title of publications in order to ensure that the maximum relationships were remained in between the literature and our research topic. Publications within the past 20 years (1995 Dec 2014) were examined. Both original research and review papers were included in our analyses.

Identified scientific publications on the effects of Se on thyroid disorders were analyzed using various different scientometric indices. These included the most active authors and institutions (highest number of publications) and the most influential authors, journals, institutions, and countries (highest number of citations). Publishing and citation trends in different years were examined and the product growth factor was calculated. Trend of science production, clustering of scientific topics and structures, and international collaborations were examined. The analytical and visualization software packages used, included Histcite, Citespace, Vosviewer, and Pajek. The Journal Citation Reports (JCR) database, an ISI information database (Thomson Reuters, New York, NY), was used to obtain the impact factors of journals with the highest number of identified studies and citations.

## Results

### Total number of published Items

**Fig. 1:**
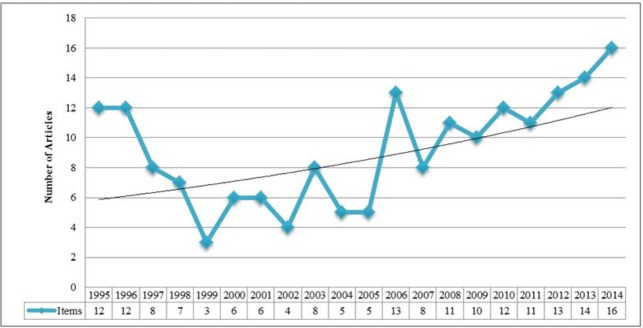
Number of articles identified with the search term, “selenium effect on thyroid disorders.” The black line represents the model that best fit the data (y = 5.6494e^0.0378x^, R^2^ = 0.2356)

Our results showed that a limited number of papers have been published on Se and the thyroid. Overall, 184 papers met our search criteria and were included in analyses. The number of scientific publications in this area was irregular. A reduction in the number of papers occurred particularly in the late 1990s, but the number of publications has been trending upwards since 2007 (Fig. 1). The highest number of papers was produced in 2014 (16 papers), and, in sum, the products growth factor was 3.78%. There was a sudden increase in scientific publication in this area in 2006.

### Total number of citations

Overall, 184 papers relevant to Se and the thyroid were cited 3815 times that were available in the ISI Web of Science database. Overall, 2975 of the 3815 total citations were self-citations. Whereby, the number of papers cited these publications were 2129 articles. The average number of citations per item was 20.73 and the total H-index was 36.

These findings are based on the citations identified from all publications available in the Web of Science database, also known as the Total of Global Citation Score (TGCS) index.

**Fig. 2:**
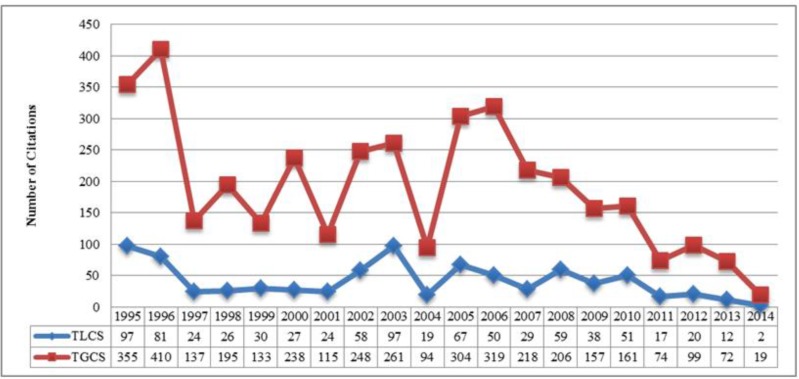
Number of citations that using the search term, “selenium effect on thyroid disorders”

However, when only citations identified from the 184 papers to themselves be used, the index is referred to as the total of the local citation score (TLCS) index. The number of citations obtained from the TLCS index was 838. The high TLCS index of a paper reveals its importance in its specialized subject. The highest number of citations obtained with the TGCS (410 citations) and TLCS (97 citations) indices was for papers published in 1996 and in 1995 and 2003, respectively (Fig. 2).

**Fig. 3:**
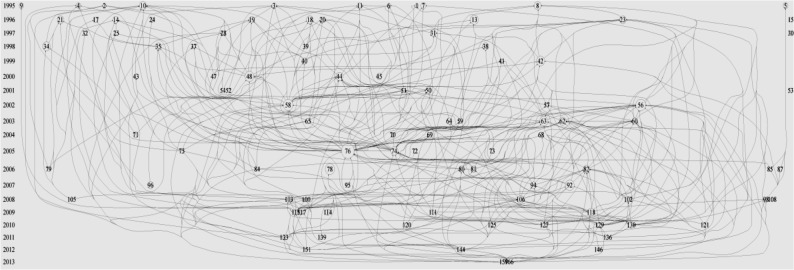
Historiogram map of research identified using the search term, “selenium effect on thyroid disorders”. Numbers in the historiogram represent the number of articles shared by these 184 papers

The historiography chart of scientific publications in this area (based on the first 100 papers identified using the TGCS) is shown in Fig. 3. Four articles with more than 100 citations are “Selenium, the thyroid, and the endocrine system”, “Selenium supplementation in patients with autoimmune thyroiditis decreases thyroid peroxidase antibodies concentrations”, “The impact of iron and selenium deficiencies on iodine and thyroid metabolism: biochemistry and relevance to public health” and “Targeted Disruption of the Type 1 Selenodeiodinase gene (Dio1) Results in Marked Changes in Thyroid Hormone Economy in Mice”.

### Distribution of publications by country and language

The publications identified on Se and the thyroid describe outcomes of studies performed in 43 countries around the world, including Germany (30 papers), the United States (27 papers), England (18 papers), and Poland (12 papers). The highest producers were defined as countries with more than 10 papers, which also included Greece, Italy, and China (11 papers each). The top 3 most influential countries, based on the number of citations identified in the TGCS index, were Germany (1272 citations), the United States (596 citations), and Belgium (401 citations). The vast majority of publications were written in English (180 of 184 papers).

### The top journals, institutions, and authors

Totally, 102 journals published scientific articles on how Se affects thyroid disorders during the defined study time period. The most productive (>5 papers) journals included *Biological Trace Element Research* (23 papers), the *European Journal of Endocrinology* (7 papers), the *Journal of Clinical Endocrinology and Metabolism* (7 papers), the *Journal of Trace Elements in Medicine and Biology* (6 papers), and *Thyroid* (6 papers). Also, Table 1 summarizes the most influential journals based on the TGCS and TLCS indices.

**Table 1:** The most influential global and local journals. Results are based on the search term, “selenium effect on thyroid disorders”

***Journal***	***Impact Factor In 2014***	***Records***	***TLCS***	***Journal***	***Impact Factor In 2014***	***Records***	***TGCS***
European Journal Of Endocrinology	4.069	7	103	Journal Of Clinical Endocrinology & Metabolism	6.209	7	294
Journal Of Clinical Endocrinology & Metabolism	6.209	7	82	Thyroid	4.493	6	287
Thyroid	4.493	6	64	European Journal Of Endocrinology	4.069	7	271
Biological Trace Element Research	1.748	23	43	Biological Trace Element Research	1.748	23	265
Endocrine Reviews	21.059	1	40	Endocrinology	4.503	5	241

TLCS = Total of local citation score, TGCS = Total of global citation score

There were 282 active educational and research institutes that published scientific articles in the area of interest. The following institutions were among the highest scientific producers (>5 papers): Charite University (8 papers, 4.3%), University of Munich (8 papers, 4.3%), Rowett Research Institute (7 papers, 3.8%), Jagiellonian University (6 papers, 3.3%), Humboldt University (6 papers, 3.3%), University of Athens (5 papers, 2.7%), and University of Edinburgh (5 papers, 2.7%). Also, Table 2 lists the most influential institutes, based on the TLCS and TGCS indices, using the “selenium impact on thyroid disorders” search term. Overall, 744 authors collaborated to produce publications on the effect of Se on thyroid disease. The 10 most influential authors, based on TLCS and TGCS indices listed in Table 3.

**Table 2:** The most influential institutions on scientific publications in the field of “selenium effect on thyroid disorders”

***Institution***	***Records***	***TLCS***
University of Munich	8	93
Humboldt University	5	76
University of Wurzburg	4	71
University of Athens	5	59
University of Edinburgh	5	49
**Institution**	**Records**	**TGCS**
University of Wurzburg	4	458
Humboldt University	5	433
University of Munich	8	281
University of Libre Bruxelles	1	197
University of Athens	5	169

TLCS=total of local citation score, TGCS=total of global citation score

**Table 3:** The most locally and globally influential authors in the field of “selenium effect on thyroid disorders” from 1995 to 2014

***Author***	***Records***	***TLCS***	***Author***	***Records***	***TGCS***
Kohrle J	14	137	Kohrle J	14	828
Gartner R	6	84	Contempre B	4	342
Contempre B	4	81	Dumont JE	4	342
Dumont JE	4	81	Gartner R	6	253
Gasnier BCH	2	58	Schomburg L	9	206
Duntas LH	3	57	Jakob F	1	197
Angstwurm MWA	2	49	Galton VA	2	169
Beckett GJ	5	49	St Germain DL	2	169
Arthur JR	8	48	Arthur JR	8	168
Krebs B	2	42	Duntas LH	3	164

TLCS = total of local citation score, TGCS = total of global citation score

### Subject trends

**Fig. 4:**
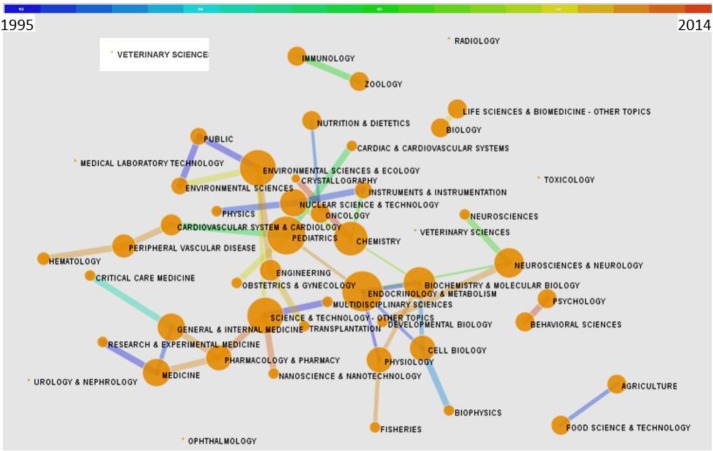
Distribution of interrelation of subject categories for papers on “selenium effect on thyroid disorders.” Dot size is proportional to the betweenness centrality. Lines between subjects indicate a relationship between those subjects and the thickness of the line indicate the strength of the link. The color of the line indicates the year in which the two related subjects frequently appeared in the same articles (see color key at top of figure)

**Fig. 5:**
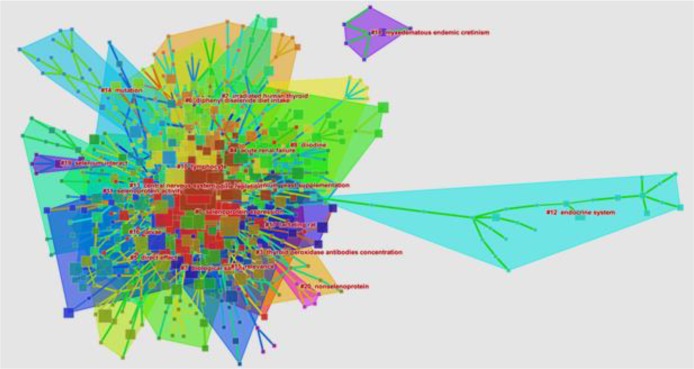
Subject clustering of articles on “selenium effect on thyroid disorders.” The subject clustering map was created with CiteSpace software. Squares represent terms in the articles, lines represent relationships between mentioned terms, and colors indicate relationship clusters (21 clusters in all) created according to the relationship and nearest of terms

**Fig. 6:**
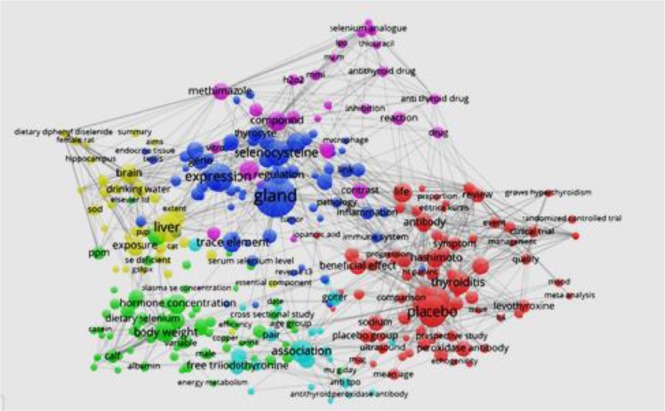
Subject distribution and clustering of them in the field of “selenium effect on thyroid disorders”

The distribution of subject trends was determined based on the ISI-Web of Science “co-occurring subject category indicator” and “page rank.” Inter-subject relationships are visually displayed in Fig. 4. Papers on the topic of interest were classified into 50 different ISI-Web of Science subject classes (Table 4). The highest number of papers were found in endocrinology and metabolism (83 papers), biochemistry and molecular biology (45 papers), and nutrition and dietetics (18 papers). In addition to the subject classification of published papers, analyzing article keywords and subject clustering is shown in Figs. 5 and 6. Subject analysis revealed that 41 scientific subjects had strong citation bursts.

**Table 4:** Subject category on “selenium effect on thyroid disorders”

***Web of Science subject category***	***Records***	***%***
Endocrinology metabolism	83	45.1
Biochemistry molecular biology	45	24.5
Nutrition dietetics	18	9.8
Veterinary sciences	10	5.4
Agriculture dairy animal science	7	3.8
Medicine research experimental	6	3.3
Medicine general internal	6	3.3
Oncology	5	2.7
Public environmental occupational health	4	2.2
Physiology	4	2.2
Multidisciplinary sciences	4	2.2
Environmental sciences	4	2.2
Chemistry multidisciplinary	4	2.2
Chemistry analytical	4	2.2

### International scientific collaboration

**Fig. 7:**
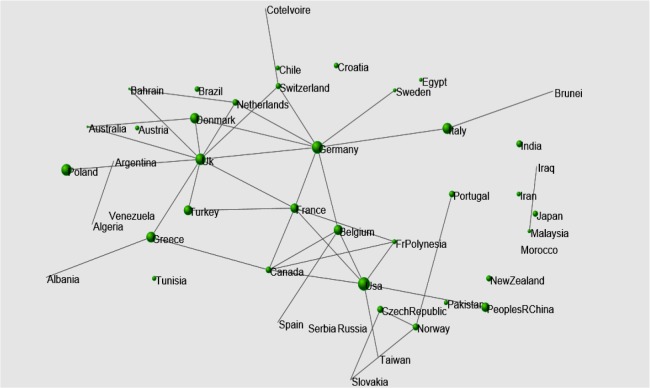
International collaborations that produced publications on the “selenium effect on thyroid disorders”. Lines represent collaborations between countries and the data point size is proportional to the number of publications produced in each country

The scientific manuscripts included in this study were the result of international collaborations among 43 countries (Fig. 7). Analyses revealed that scientists and physicians in the following countries had the greatest number of collaborations: England, Germany, France, and the United States.

## Discussion

The number of publications, the number of citations, and publication trends were examined with our analyses regarding impact of Se on thyroid disorders. Overall, the number of articles on how Se affects thyroid disorders is growing. Interestingly, there was a sudden increase in the number of articles published from 2005 (5 articles) to 2006 (13 articles). This increase may have occurred for several reasons, including emergence of a new disease topic. Article citations in this subject area were examined using two total H-index of all articles and the average number of citations per paper. Similar to the number of publications, the number of citations varied in an irregular fashion. Papers published in 1996 were cited more often than papers published in other years, as shown by the TGCS index. Because no prior studies of this kind with this setting have been performed in this field, we were unable to compare our results with other similar studies.

Scientometric analyses was used to examine the distribution of male and female patients with thyroid cancer around the world. Their analysis used science citation index (1991–2010) to specifically examine gender in thyroid cancer publication output. Overall, 380 publications were published by scientists on gender and thyroid cancer. The average number of studies published per year was 10.7% and highest in 2006 and 2007 (41 articles). The number of publications was highest for thyroid (273 publication), cancer (131 publications), and carcinoma (112 publications) (13). Additionally, Qin (14) published an article that used bibliometric research methods to examine publications on the antioxidant effect of Se published between 2002 and 2011 and were indexed in the SCI database. Their findings provided information on the antioxidant effect of Se and identified research hot spots in the field (14). A scientometric analysis was published on endocrine and diabetes research in SAARC (South Asian Association for Regional Cooperation) Countries which identified trends of related research activity and more influential countries, journals as well as authors in mentioned field (15).

A 2014 study was examined thyroid research and practice. This study analyzed Thyroid Research and Practice journal articles published between 2006 and 2013. The total number of publications, the type of articles, and the names of authors were examined across various subspecialties. Publications were also classified as having Indian or foreign and academic or non-academic origins, determined using first author’s affiliation. During the study period, 23 main issues and 1 supplementary issue were published and included 210 publications. The majorities of manuscripts published were original articles and had widespread coverage across all thyroidology subspecialties. Articles were most often related to clinical thyroidology and originated from academic institutions. Additionally, publications were most often from India with more contribution from Kerala, but articles from other countries were increasing in number (16).

Countries that actively published scientific papers on Se and thyroid disorders included Germany, The United States, England, Italy, Poland, and Greece. These countries were the most productive and had the greatest influence on the topic. The journals that published most often and that were most influential included *Biological Trace Element Research*, the *European Journal of Endocrinology*, the *Journal of Clinical Endocrinology and Metabolism*, and *Thyroid*. The most active and effective institutions were the University of Munich, Humboldt University, and the University of Athens. The most influential authors according to TGCS and TLCs indices were Kohrle J., Gartner R., Contempre B., Dumon JE., Duntas JE., and Arthur J. R.

Publications were most often categorized into the following subjects: “Endocrinology and Metabolism,” “Biochemistry and Molecular Biology,” and “Nutrition and Dietetics.” Categorization mapping (Fig. 4), showed new aspects of how these subjects were interrelated during the publication years examined.

Well-designed studies in special conditions such as necessity of selenium in autoimmuine thyroid disease for instance in pregnant women are needed. These studies may shed light on further research in this field but in special conditions (17–19).

Using wider databases will formulate valid outcomes (20). Our study was limited since analyses only examined publications indexed in the ISI Web of Science database. The study also had much strength, including the long study period and its thorough investigation of the impact of publications, countries, organizations, and international relationships. Additionally, the inclusion of systematic citation analyses and mapping fields make our findings particularly significant.

## Conclusion

This study reveals new aspects of intersubject relationships present in the literature. Leading scientific issues on the effect of Se on thyroid disorders were determined by examining impact extent and study citation sets of published studies. More specifically, the publication impact, organization impact, systematic citation search outcomes, mapping fields, and indicator construction were examined to better understand what policies and management decisions were launched. Our results indicate the need for similar studies to be performed within the next five years to continue our understanding of scientific publication trends in this field.

## Ethical considerations

Ethical issues (Including plagiarism, informed consent, misconduct, data fabrication and/or falsification, double publication and/or submission, redundancy, etc.) have been completely observed by the authors.
